# Effects of aerobic exercise on late effects and quality of life in long-term breast cancer survivors: a randomized controlled trial

**DOI:** 10.1093/jncics/pkaf102

**Published:** 2025-10-21

**Authors:** Sara Hassing Johansen, Kristin V Reinertsen, Torbjørn Wisløff, Mali Sæter, Sebastian Imre Sarvari, Cecilie E Kiserud, Nora Strandos, Elisabeth Edvardsen, May Grydeland, Tormod S Nilsen, Lene Thorsen

**Affiliations:** Department of Physical Performance, The Norwegian School of Sport Sciences, Oslo, Norway; Department of Oncology, Division of Cancer Medicine, Oslo, Norway; Institute of Clinical Medicine, Faculty of Medicine, University of Oslo, Oslo, Norway; Health Services Research Unit, Akershus University Hospital, Lørenskog, Norway; Institute of Clinical Medicine, Faculty of Medicine, University of Oslo, Oslo, Norway; ProCardio Center for Innovation, Department of Cardiology, Oslo University Hospital, Oslo, Norway; ProCardio Center for Innovation, Department of Cardiology, Oslo University Hospital, Oslo, Norway; Department of Oncology, Division of Cancer Medicine, Oslo, Norway; Department of Physical Performance, The Norwegian School of Sport Sciences, Oslo, Norway; Department of Physical Performance, The Norwegian School of Sport Sciences, Oslo, Norway; Department of Pulmonary Medicine, Oslo University Hospital, Oslo, Norway; Department of Physical Performance, The Norwegian School of Sport Sciences, Oslo, Norway; Department of Physical Performance, The Norwegian School of Sport Sciences, Oslo, Norway; Department of Oncology, Division of Cancer Medicine, Oslo, Norway; Department for Clinical Service, Division of Cancer Medicine, Oslo University Hospital, Oslo, Norway

## Abstract

**Background:**

Breast cancer survivors (BCSs) are at increased risk of late effects. While research has reported positive effects of exercise therapy on fatigue and health-related quality of life (HRQoL) among short-term BCSs, evidence in long-term survivors remains scarce.

**Methods:**

The CAUSE (CArdiovascUlar Survivors Exercise) trial was a 2-armed randomized controlled trial. Long-term BCSs were assigned to 5 months of thrice-weekly supervised aerobic exercise or usual care. Late effects and HRQoL were assessed by Chalder Fatigue Questionnaire, European Organization for Research and Treatment of Cancer QLQ-BR23 and QLQ-C30 questionnaires, and Scale for Chemotherapy-Induced Long-term Neurotoxicity at baseline (T0), post-intervention (T1), and 1-year follow-up (T2).

**Results:**

In total, 140 BCSs (mean age 59.0 ± 6.4 years, 11 ± 1 years post-treatment) were included. Loss to follow-up at T1 was 6% and 19% in the exercise- and usual care group, respectively. From T0 to T1, the exercise group significantly improved total fatigue (between groups mean difference [MD] = –3.0, *P *< .001), body image (MD = 6.7, *P *= .043), physical- (MD = 3.2, *P* ≤ .001), role- (MD = 9.6, *P *= .019), and cognitive function (MD = 3.4, *P *= .038), insomnia (MD = –9.0, *P *= .017), and global health/QoL (MD = 5.3, *P ≤* .001) compared to usual care. The exercise benefits were more pronounced in BCSs experiencing versus not experiencing late effects at baseline. At 1-year follow-up, most improvements regressed toward baseline values.

**Conclusion:**

Aerobic exercise significantly improves fatigue, body image, physical-, role-, and cognitive function, insomnia, and HRQoL in long-term BCSs. These findings suggest that exercise therapy should be a core component of managing late effects and enhancing HRQoL in long-term BCSs.

**Clinical trial registration:**

URL: https://www.clinicaltrials.gov/. Registration number: NCT04307407

## Introduction

Breast cancer is the most common malignancy among women worldwide.^1^ Due to advancements in diagnostics and treatments, the 5-year survival rate has surpassed 90% in several Western countries.^2^^,^^3^ However, breast cancer survivors (BCSs) are at increased risk of several late effects, ie, complaints or health problems related to the cancer or the cancer therapy that might persist or emerge years after treatment, such as fatigue, insomnia, cognitive impairments, emotional symptoms, and sexual dysfunction.^4^^,^^5^ A recent cross-sectional study among ∼1350 long-term BCSs (ie, >5 years post-treatment) demonstrated that almost 50% had moderate to high symptom burden negatively affecting their general functioning.^6^ Nearly 45% reported pain, 45% reported cognitive dysfunction, 34% reported sleep disturbances, and 32% suffered from chronic fatigue.^6^ These findings are consistent with previous longitudinal studies on fatigue and insomnia among long-term BCSs.^7^^,^^8^ Thus, as the population of BCSs continues to grow, effectively managing these late effects is a crucial aspect of survivorship care.

Exercise therapy has emerged as an effective strategy to improve overall health in cancer survivors.^9-11^ While previous studies have demonstrated beneficial effects of exercise therapy on fatigue, mental health, and HRQoL during and shortly after active treatment,^12-18^ few trials have investigated the effects of exercise therapy on late effects in long-term BCSs.^10^^,^^19^ Therefore, the aim of this study was to evaluate the effects of supervised aerobic exercise on late effects and HRQoL in long-term BCSs and to compare the effects of aerobic exercise among BCSs experiencing late effects at baseline and BCSs not experiencing late effects at baseline.

## Methods

The design and methods for the CAUSE (CArdiovascUlar Survivors Exercise) trial have been reported previously.^20^ In brief, the CAUSE trial is a 2-armed, phase II randomized controlled trial comparing the effects of aerobic exercise, aimed at increasing cardiorespiratory fitness, to usual care in long-term female BCSs. Here, we present secondary analyses to evaluate the effects of aerobic exercise on late effects and HRQoL. The CAUSE trial was conducted according to the Helsinki Declaration, approved by the Regional Committees for Medical and Health Research Ethics (REC) (2019/1318), and preregistered on clinicaltrials.gov (NCT04307407). The trial also adhered to the CONSORT statement for non-pharmacological trials (Table S1).^21^

### Participants

Epirubicin-treated female BCSs diagnosed with stage Il-III breast cancer at the age of ≤60 years between 2008 and 2012 were identified through the Cancer Registry of Norway. Identified BCSs were invited through postal mail and screened for eligibility by phone. Exclusion criteria were ≥90 minutes of self-reported exercise per week, former Trastuzumab treatment, recurrent breast cancer or presence of other malignancies (except for basal cell carcinomas), pacemaker therapy, previous major cardiac surgery, recent or uncontrolled cardiovascular disease, health conditions that by self-evaluation restricted adherence to study protocol, medical contraindications to exercise, or participation in other exercise trials. Recent or uncontrolled cardiovascular disease was defined as conditions newly diagnosed within the last 3 months or not effectively managed, posing a risk of further cardiovascular complications (eg, heart failure, arrhythmias, moderate to severe valve disease, coronary artery disease, or symptoms such as chest pain, shortness of breath, or syncope).

### Randomization and blinding

Eligible BCSs were randomly assigned in a 1:1 ratio to aerobic exercise or usual care without any stratification factors. The randomization sequence remained concealed until baseline assessments were completed. At post-intervention, participants and assessors were aware of group allocations but remained blinded to baseline results.

### Exercise intervention

The 5-month aerobic exercise program consisted of thrice-weekly supervised treadmill walking and/or running sessions following a non-linear progression model. The exercise prescription included both continuous sessions at low to moderate exercise intensities (ie, 25–40-minute duration, 60-85% peak heart rate [HR_peak_]) and high-intensity interval sessions (ie 16-32-minute duration, 85-97% of HR_peak_). The intensity was individually tailored to each participant’s HR_peak_ measured during a cardiopulmonary exercise test pre-intervention. Ratings of perceived exertion using the Borg Scale^22^ and pre-defined HR-zones were used to adjust the exercise intensity (S2).^23^

Participants in usual care were encouraged to maintain their usual activity level during the intervention period.

### Outcomes

All outcomes were assessed at baseline (T0), 5 months post-intervention (T1), and at 1-year post-intervention (T2).

#### Late effects

Fatigue was assessed by the Chalder Fatigue Questionnaire (FQ).^24^ FQ consists of 11 items, assessing physical fatigue (7 items), mental fatigue (4 items), and total fatigue (sum of physical and mental fatigue). Each item was rated on a 4-point Likert scale from 0 to 3 (0 = “less or better than usual/not at all” to 3 = “a lot more than usual”), with higher scores reflecting more fatigue. The summarized physical fatigue score ranged from 0 to 21, the mental fatigue score from 0 to 12, and the total fatigue score from 0 to 33. In addition, a separate item assessed fatigue duration (<1 week, <3 months, 3-6 months, and ≥6 months).

Body image, sexual functioning, future perspective, and breast- and arm symptoms were assessed by the European Organization for Research and Treatment of Cancer (EORTC) BC-specific module (EORTC QLQ-BR23).^25^ Items were rated on a 4-point Likert scale from 1 to 4 (1 = “not at all” to 4 = “very much”), and transformed to a 0 to 100 scale, according to established EORTC scoring manuals.^26^ Higher transformed scores on the body image (4 items), sexual functioning (2 items), and future perspective (1 item) scales indicated a better body image, sexual functioning, and future perspective, whereas higher scores on breast symptoms (4 items) and/or arm symptoms (3 items) indicated higher symptom levels.

Presence of neuropathy in hands and/or feet was assessed by 2 items from the Scale for Chemotherapy-Induced Long-term Neurotoxicity (SCIN).^27^ Each item was rated from 0 (not at all) to 3 (very much), giving a total score ranging from 0 to 6.

#### Health-related quality of life aspects

HRQoL aspects, covering functioning, symptoms, problems, and global health/QoL were measured by EORTC QLQ-C30 version 3.^28^ The EORTC QLQ-C30 includes 5 functional scales (physical-, emotional-, role-, social-, and cognitive functioning), 3 symptom scales (fatigue, nausea and vomiting, and pain), 6 single items (dyspnea, insomnia, appetite loss, constipation, diarrhea, and financial difficulties), and a global health status/QoL scale. All items were rated on a 4-point Likert scale from 1 to 4 (1 = “not at all” to 4 = “very much”), except the 2 global health/QoL items, which were rated from 1 = “very poor” to 7 = “excellent”. Scales were transformed to a 0 to 100 scale, according to established EORTC scoring manuals.^26^ Higher scores on the functional scales and global health/QoL scale indicated a higher level of functioning and higher global health/QoL, and a higher score on the symptom scales/single items indicated a higher symptom level/problem.

#### Definition of BCSs experiencing late effects at baseline

To identify BCSs experiencing late effects (eg, functional impairments, symptoms, and/or problems) at baseline, we used the following cut-offs:

1. Fatigue: BCSs with chronic fatigue, defined as a total sum score ≥4 on the dichotomized FQ responses, where ratings of 0 or 1 were scored as 0, and ratings of 2 or 3 were scored as 1, for 6 months or longer.^24^
2. Body image concern, breast and/or arm symptoms: BCSs who reported “3 = much” or “4 = very much” on ≥1 item on the body image scale, the breast- and/or the arm symptom scales on the EORTC QLQ-BR23, respectively.
3. Sexual dysfunction: BCSs who answered “not at all” (score = 1) to both items (“sexual interest” and “sexual activity”) on the EORTC QLQ-BR23, resulting in a transformed score of 0.
4. Neuropathy: BCSs who reported a total score ≥4^6^ on the SCIN.
5. HRQoL aspects including functional impairments, and/or high burden of symptoms/or problems: We used the established thresholds for clinical importance for the EORTC QLQ-C30.^29^

#### Background variables

Living status, education level, and daily smoking habits were measured by similar questions as in the HUNT4 study (the Trøndelag Health Study).^30^ Height and weight were measured using a stadiometer (SECA 213, Hamburg, Germany). Body mass index (BMI) was calculated from measured height and weight (kg/m^2^). Self-reported moderate to vigorous physical activity was assessed by a modified Godin-Shepard Leisure-Time Physical Activity Questionnaire.^31^ Cancer-related variables (age at diagnosis, years since epirubicin discontinuation and dosage, and treatment modality) were obtained from medical records.

### Statistical analysis

The power calculation, based on peak oxygen consumption, determined that 63 BCSs were required in each group to achieve a 90% power at a 5% significance level.^20^ To account for an anticipated 10% dropout rate, 70 BCSs were included per group.

Baseline characteristics are presented as numbers and percentages, mean and standard deviation (SD), or median and interquartile range (IQR). Analyses were conducted using the intention-to-treat principle, and missing data were handled using the last observation carried forward. Changes from T0 to T1 and from T0 to T2 were estimated individually, using the mean change within each group to estimate differences between groups. For the EORTC questionnaires, missing items were handled according to the scoring manuals.^26^

Due to the non-normality of residuals in the outcome variables, and that variables naturally have a lower and upper boundary, we performed beta regression to evaluate the effect of aerobic exercise.^32^ The values for the outcome variables at T1 and T2 were normalized to a scale between 0 and 1, respectively, controlling for the T0 value of the outcome variable. For all analyses with beta regression, we used a logit link function, which provides the opportunity to show results as odds ratios.^33^ For the dichotomized neuropathy variable (high vs low level of neuropathy), we performed logistic regression. Sub-group analyses were performed using the described cutoffs for BCSs experiencing late effects at baseline.

Results are presented with odds ratio (OR), and 95% confidence intervals (95% CI), with statistical significance set at *P *< .05. All analyses were performed in RStudio version 2023.06.0 using R version 4.4.1.

## Results

**Figure 1.**
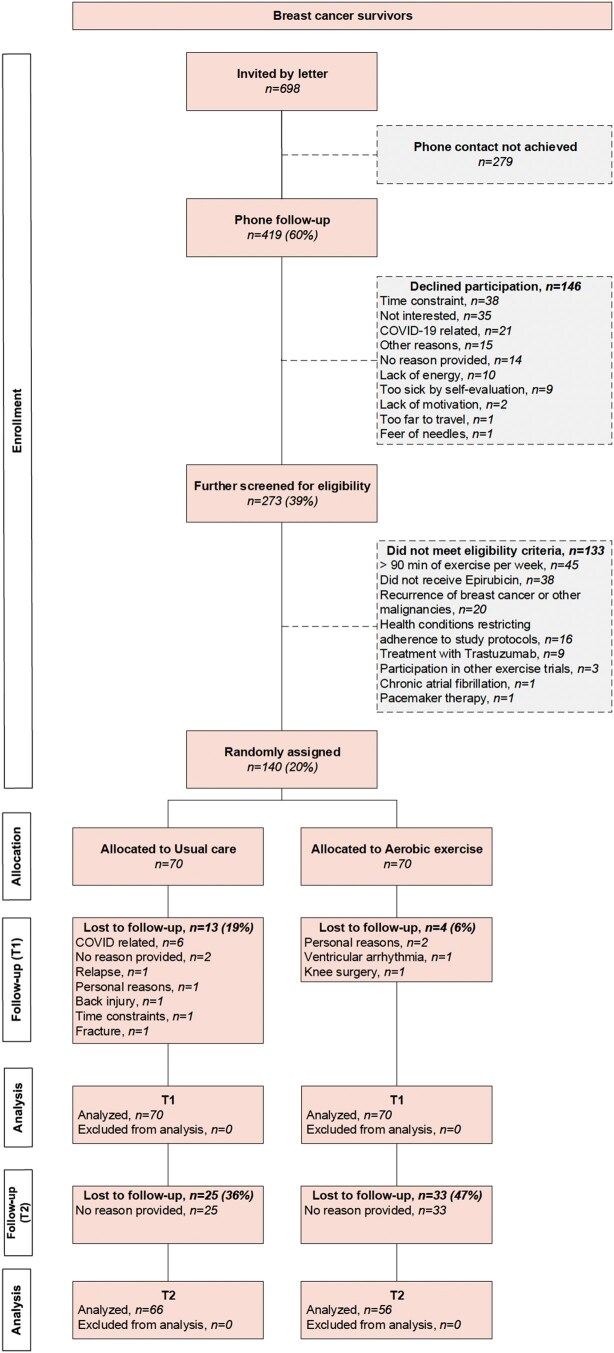
CONSORT (Consolidated Standards of Reporting Trials) flow diagram for inclusion of participants. The figure follows the Consolidated Standards of Reporting Trials and details the numbers of participants assessed for eligibility, enrolled, allocated to groups (aerobic exercise or usual care), loss to follow-up, and included in the analysis at T1 and T2.

Between October 2020 and August 2022, 698 BCSs were found eligible and were invited to participate. A total of 140 (20%) consented and were randomized to the exercise group (*n* = 70) or the usual care group (*n* = 70). Loss to follow-up from T0 to T1 was 6% (*n* = 4) and 19% (*n* = 13) in the exercise and usual care group, respectively. At T2, 36% (*n* = 25) in the exercise group and 47% (*n* = 33) in the usual care group did not respond to the questionnaire (Figure 1, Table S3). Mean age at T0 was 59.0 ± 6.4 years, and the average time since epirubicin discontinuation was 11 ± 1 years (Table 1). The mean exercise intervention attendance and adherence was 78% and 72%, respectively.

**Table 1.** Baseline characteristics of the participants.

	Total BCSs (*n* = 140)	Exercise group (*n* = 70)	Usual care group (*n* = 70)
Socio-demographic			
Age at survey, y	59.0 ± 6.4	58.6 ± 6.0	59.4 ± 6.8
Living with partner, *n* (%)			
Yes	99 (71)	51 (73)	48 (69)
No	41 (29)	19 (27)	22 (31)
Education level >13 years, *n* (%)			
Yes	93 (66)	44 (63)	49 (70)
No	46 (33)	26 (37)	20 (29)
Lifestyle and health			
Height, cm	168.0 ± 5.8	168.0 ± 5.4	168.1 ± 6.3
Body weight, kg	76.0 ± 13.5	75.5 ± 13.0	76.5 ± 14.1
Body mass index, kg/m^2^	26.9 ± 4.5	26.7 ± 4.4	27.1 ± 4.6
Daily smokers, *n* (%)	6 (4)	3 (4)	3 (4)
Self-reported moderate to vigorous physical activity, minutes/week	95.9 ± 142.9	94.7 ± 132.8	97.2 ± 153.4
Cancer-related variables			
Age at diagnosis, y	48.0 ± 6.2	47 ± 5.9	48 ± 7.0
Years since Epirubicin discontinuation, y	11 ± 1	11 ± 1	11 ± 1
Hormone receptor-positive, *n* (%)	109 (78)	53 (76)	56 (80)
Cumulative Epirubicin dose, mg · m^-2^^a^^,^^b^	357 [243-366]	358 [247-365]	353 [241-367]
Taxane therapy,^a^ *n* (%)	69 (50)	32 (46)	37 (54)
Radiotherapy, *n* (%)	119 (85)	56 (80)	63 (90)
Left-sided radiotherapy, *n* (%)	72 (51)	41 (59)	31 (44)
Endocrine therapy	110 (79)	54 (77)	56 (80)
Tamoxifen only, *n* (%)	54 (39)	28 (40)	26 (37)
Aromatase inhibitor only, *n* (%)	20 (14)	10 (14)	10 (14)
Tamoxifen+ aromatase inhibitor, *n* (%)	36 (26)	16 (23)	20 (29)

Data expressed as *n* (%), mean ± SD or median [IQR].

Abbreviation: BCSs = breast cancer survivors.

a Details on chemotherapy regimen are missing from 1 patient.

b Administered in combination with 5-fluorouracil and cyclophosphamide.

### Effect of aerobic exercise on fatigue

From T0 to T1, the mean difference (MD) in change between groups was –3.0 points for total fatigue (OR = 0.58, 95% CI = 0.46 to 0.74, *P *< .001), –2.3 points for physical fatigue (OR = 0.52, 95% CI = 0.41 to 0.67, *P *< .001), and –0.8 points for mental fatigue (OR = 0.67, 95% CI = 0.53 to 0.84, *P *< .001) (Table 2).

**Table 2.** Effect of aerobic exercise on fatigue, other late effects, and health-related quality of life in BCSs compared to usual care.

					Within group differences		Between group differences T0 to T1^a^			Between group differences T0 to T2^a^		
	Group	T0	T1	T2	Change T0-T1	Change T0-T2	Change T0-T1	Odds ratio (95% CI)	*P*	Change T0-T2	Odds ratio (95% CI)	*P*
Fatigue												
Total fatigue	Exercise	13.4 ± 5.3	10.3 ± 4.8	12.0 ± 5.2	−3.2 ± 6.3	−1.7 ± 7.3	−3.0	0.58 (0.46 to 0.74)	<.001	−2.1	0.82 (0.62 to 1.08)	.150
	Usual care	13.2 ± 4.2	13.1 ± 4.1	13.7 ± 5.1	−0.1 ± 4.1	0.4 ± 4.4						
Physical fatigue	Exercise	8.4 ± 3.6	6.2 ± 3.5	7.6 ± 3.7	−2.2 ± 4.4	−0.9 ± 5.0	−2.3	0.52 (0.41 to 0.67)	<.001	−1.1	0.84 (0.62 to 1.13)	.258
	Usual care	8.3 ± 2.9	8.4 ± 3.0	8.5 ± 3.5	0.1 ± 3.0	0.2 ± 3.1						
Mental fatigue	Exercise	5.1 ± 1.9	4.1 ± 1.6	4.4 ± 2.0	−1.0 ± 2.2	−0.8 ± 2.7	−0.8	0.67 (0.53 to 0.84)	<.001	−1.0	0.68 (0.48 to 0.96)	.030
	Usual care	4.9 ± 1.6	4.7 ± 1.5	5.2 ± 1.9	−0.2 ± 1.4	0.2 ± 1.7						
Other late effects												
Body image	Exercise	72.8 ± 24.5	80.5 ± 19.5	78.5 ± 22.8	7.7 ± 15.5	5.3 ± 15.7	6.7	1.40 (1.01 to 1.93)	.043	3.5	1.01 (0.63 to 1.61)	.983
	Usual care	78.4 ± 20.0	79.3 ± 19.4	79.3 ± 20.1	1.0 ± 16.6	1.8 ± 19.0						
Sexual functioning	Exercise	23.3 ± 24.3	24.5 ± 25.7	23.4 ± 26.3	1.2 ± 18.5	0.3 ± 20.5	0.5	0.69 (0.49 to 0.98)	.038	0.3	0.91 (0.56 to 1.48)	.698
	Usual care	25.8 ± 18.9	27.1 ± 20.1	24.3 ± 19.4	0.7 ± 13.2	0 ± 15.9						
Future perspective	Exercise	69.1 ± 21.6	76.8 ± 21.6	72.9 ± 22.0	7.7 ± 20.7	3.1 ± 28.3	1.0	1.25 (0.85 to 1.83)	.260	−2.2	1.22 (0.67 to 2.22)	.509
	Usual care	62.3 ± 29.1	69.1 ± 23.8	65.5 ± 22.0	6.8 ± 23.3	5.4 ± 23.6						
Breast symptoms	Exercise	10.4 ± 15.0	9.2 ± 13.3	10.3 ± 13.8	−1.2 ± 10.6	0.5 ± 12.9	−1.6	0.78 (0.56 to 1.08)	.132	−1.4	0.90 (0.57 to 1.44)	.668
	Usual care	11.9 ± 15.4	12.5 ± 15.1	14.2 ± 15.0	0.4 ± 10.1	1.9 ± 12.8						
Arm symptoms	Exercise	16.7 ± 22.4	15.4 ± 21.1	19.4 ± 22.5	−1.3 ± 19.0	3.4 ± 20.8	−3.5	0.72 (0.51 to 1.02)	.067	−0.8	1.05 (0.63 to 1.74)	.857
	Usual care	12.1 ± 17.3	15.1 ± 20.6	17.9 ± 24.1	2.3 ± 11.0	4.2 ± 16.9						
Neuropathy^b^	Exercise	12 (17)	7 (10)	7 (11)	−5	−5	0	0.44 (0.09 to 1.87)	.280	0	0.40 (0.09 to 1.87)	.310
	Usual care	12 (17)	7 (17)	6 (11)	−5	−5						
HRQoL												
Physical functioning	Exercise	91.0 ± 12.2	92.3 ± 11.4	90.1 ± 11.5	1.3 ± 7.5	−0.7 ± 7.5	3.2	1.72 (1.25 to 2.35)	<.001	1.0	0.89 (0.58 to 1.38)	.610
	Usual care	88.1 ± 13.6	86.2 ± 14.0	86.8 ± 16.8	−1.9 ± 7.7	−1.7 ± 9.1						
Emotional functioning	Exercise	89.3 ± 12.0	91.1 ± 12.1	89.9 ± 14.0	1.7 ± 12.7	−0.5 ± 14.6	1.5	1.13 (0.80 to 1.60)	.482	−0.3	1.05 (0.66 to 1.69)	.826
	Usual care	90.4 ± 12.5	90.6 ± 16.4	90.2 ± 13.2	0.2 ± 11.5	−0.1 ± 12.5						
Role functioning	Exercise	85.0 ± 23.1	91.0 ± 16.5	85.4 ± 23.5	6.0 ± 17.7	1.0 ± 26.3	9.6	1.53 (1.07 to 2.19)	.019	5.9	1.30 (0.78 to 2.18)	.314
	Usual care	86.3 ± 22.1	82.6 ± 25.0	82.7 ± 25.4	−3.7 ± 17.2	−4.8 ± 21.0						
Social functioning	Exercise	81.0 ± 25.7	85.5 ± 22.1	82.6 ± 23.8	4.5 ± 24.6	1.3 ± 26.8	3.6	1.19 (0.83 to 1.73)	.343	1.0	1.18 (0.71 to 1.96)	.523
	Usual care	81.9 ± 25.4	82.9 ± 24.2	83.6 ± 24.9	1.0 ± 15.6	0.3 ± 22.1						
Cognitive functioning	Exercise	77.5 ± 24.7	82.9 ± 20.0	83.8 ± 22.4	4.8 ± 15.5	5.9 ± 19.4	3.4	1.44 (1.02 to 2.03)	.038	9.8	1.59 (0.98 to 2.61)	.063
	Usual care	80.9 ± 21.2	82.4 ± 19.7	77.0 ± 23.7	1.5 ± 11.1	−3.9 ± 18.7						
Fatigue	Exercise	27.2 ± 25.5	23.3 ± 23.1	28.8 ± 23.8	−3.9 ± 17.4	1.4 ± 19.4	−4.0	0.62 (0.44 to 0.88)	.007	−1.0	1.02 (0.62 to 1.67)	.937
	Usual care	27.6 ± 23.2	27.8 ± 23.0	29.7 ± 24.7	0.2 ± 15.8	2.4 ± 18.3						
Nausea and vomiting	Exercise	2.14 ± 6.29	1.43 ± 4.70	2.52 ± 7.30	−0.7 ± 6.6	0.8 ± 8.0	−1.0	0.85 (0.61 to 1.18)	.338	0.2	1.01 (0.64 to 1.59)	.965
	Usual care	2.17 ± 8.04	2.42 ± 7.71	2.38 ± 7.41	0.2 ± 4.5	0.6 ± 7.1						
Pain	Exercise	16.2 ± 20.8	14.8 ± 19.4	21.5 ± 24.4	−1.4 ± 18.1	5.3 ± 24.7	−3.1	0.76 (0.53 to 1.09)	.134	1.4	0.81 (0.48 to 1.35)	.414
	Usual care	22.5 ± 27.8	24.2 ± 26.4	26.8 ± 26.9	1.7 ± 17.9	3.9 ± 16.2						
Dyspnea	Exercise	11.0 ± 19.4	7.62 ± 15.2	8.59 ± 17.8	−3.3 ± 16.2	−2.5 ± 19.7	−6.8	0.74 (0.52 to 1.05)	.092	−6.2	0.81 (0.50 to 1.31)	.390
	Usual care	7.35 ± 17.1	10.8 ± 17.7	9.70 ± 17.8	3.4 ± 14.3	3.6 ± 15.3						
Insomnia	Exercise	32.4 ± 30.0	21.9 ± 25.3	23.7 ± 27.3	−10.5 ± 24.4	−7.6 ± 26.7	−9.0	0.61 (0.41 to 0.92)	.017	−11.1	0.45 (0.25 to 0.83)	.010
	Usual care	34.8 ± 31.0	33.3 ± 34.3	41.7 ± 33.2	−1.5 ± 25.2	3.6 ± 34.6						
Appetite loss	Exercise	5.80 ± 16.12	3.38 ± 10.1	5.13 ± 14.7	−2.4 ± 13.2	−1.0 ± 17.6	−1.9	0.90 (0.65 to 1.27)	.560	−1.0	1.09 (0.68 to 1.74)	.732
	Usual care	3.38 ± 11.63	2.90 ± 9.46	2.98 ± 11.5	−0.5 ± 7.0	0 ± 11.0						
Constipation	Exercise	12.4 ± 22.8	9.52 ± 17.2	11.1 ± 18.8	−2.9 ± 18.6	1.0 ± 22.6	−3.3	0.81 (0.56 to 1.17)	.261	−3.4	0.86 (0.51 to 1.45)	.565
	Usual care	13.5 ± 22.4	14.0 ± 25.8	14.9 ± 25.4	0.5 ± 16.7	2.4 ± 22.8						
Diarrhea	Exercise	9.05 ± 18.8	9.52 ± 17.2	9.09 ± 16.06	0.5 ± 19.2	1.0 ± 19.4	1.5	0.98 (0.69 to 1.41)	.924	−0.2	1.05 (0.63 to 1.75)	.861
	Usual care	11.4 ± 17.9	10.4 ± 19.4	12.7 ± 23.6	−1.0 ± 14.2	1.2 ± 18.1						
Financial difficulties	Exercise	7.62 ± 18.1	6.67 ± 17.6	5.05 ± 13.4	−1.0 ± 16.0	−2.0 ± 14.2	−0.5	0.85 (0.60 to 1.20)	.359	−0.8	0.97 (0.61 to 1.55)	.912
	Usual care	7.72 ± 22.2	7.25 ± 21.3	5.36 ± 15.3	−0.5 ± 9.0	−1.2 ± 16.8						
Global health/QoL	Exercise	75.5 ± 17.0	78.9 ± 17.5	74.9 ± 21.5	3.4 ± 18.0	−1.1 ± 23.6	5.3	1.76 (1.26 to 2.46)	<.001	2.3	1.69 (1.06 to 2.69)	.028
	Usual care	75.6 ± 16.6	73.8 ± 19.7	72.6 ± 21.3	−1.8 ± 14.0	−3.4 ± 17.6						

Data are expressed as mean ± SD or *n* (%). *P*-value is derived from a beta regression.

Abbreviations: HRQoL = health-related quality of life; QoL = quality of life; T0 = baseline; T1 = post-intervention; T2 = 1-year follow-up.

a Analyses were adjusted for baseline values of the respective outcome variable.

b *P*-value is derived from logistic regression showing participants with a high degree of neuropathy.

From T0 to T2, mental fatigue decreased by –0.8 ± 2.7 points in the exercise group compared to an increase of 0.2 ± 1.7 points in the usual care group (MD = –1.0, OR = 0.68, 95% CI = 0.48 to 0.96, *P *= .030). No significant differences between groups were observed for total fatigue or physical fatigue (Table 2).

### Effect of aerobic exercise on other late effects

From T0 to T1, the exercise group reported improved body image with 7.7 ± 15.5 points compared to 1.0 ± 16.6 points among those in the usual care group (MD = 6.7, OR = 1.40, 95% CI = 1.01 to 1.93, *P *= .043). Sexual functioning improved by 1.2 ± 18.5 points in the exercise group compared to 0.7 ± 13.2 points in usual care (MD = 0.5, OR = 0.69, 95% CI = 0.49 to 0.98, *P *= .038) (Table 2). No significant differences were observed for the other late effects from T0 to T1 or from T0 to T2 (Table 2).

### Effect of aerobic exercise on HRQoL

Compared to the usual care group, the exercise group significantly improved physical- (MD = 3.2, OR = 1.72, 95% CI = 1.25 to 2.35, *P ≤* .001), role- (MD = 9.6, OR = 1.53, 95% CI = 1.07 to 2.19, *P *= .019), and cognitive- (MD = 3.4, OR = 1.44, 95% CI = 1.02 to 2.03, *P *= .038) functioning, and global health/QoL (MD = 5.3, OR = 1.76, 95% CI = 1.26 to 2.46, *P *= .001) from T0 to T1, and reduced their level of total fatigue (MD = –4.0, OR = 0.62, 95% CI = 0.44 to 0.88, *P *= .007), and insomnia (MD = –9.0, OR = 0.61, 95% CI = 0.41 to 0.92, *P *= .017) (Table 2). Compared to T0, insomnia remained significantly lower (MD = –11.1, OR = 0.45, 95% CI = 0.25 to 0.83, *P *= .010), and global health/QoL significantly higher (MD = 2.3, OR = 1.69, 95% CI = 1.06 to 2.69, *P *= .028) at T2 in the exercise group compared to usual care (Table 2).

### Effects of aerobic exercise among BCSs experiencing late effects at baseline

BCSs experiencing late effects at T0 (ie, chronic fatigue, arm symptoms, physical-, social-, and cognitive functioning impairments, fatigue, nausea and vomiting, dyspnea, and/or constipation) experienced greater effects of the exercise intervention at T1 compared to BCSs not experiencing such late effects at T0 (Table 3, Table S4).

**Table 3.** Sub-group analysis on the effect of aerobic exercise compared to usual care in participants experiencing late effects at T0 compared to those without such complaints.

	BCSs experiencing late effects at T0						BCSs not experiencing late effects at T0					
	*n*	Exercise group T0-T1	*n*	Usual care T0-T1	Odds ratio (95% CI)^a^	*P*	*n*	Exercise group T0-T1	*n*	Usual care T0-T1	Odds ratio (95% CI)^a^	*P*
Fatigue												
Total fatigue	14	−7.2 ± 11.0	15	−2.2 ± 6.6	0.48 (0.33 to 0.69)	<.001	56	2.1 ± 3.9	54	0.4 ± 2.8	0.76 (0.44 to 1.32)	.329
Physical fatigue	14	−4.8 ± 7.4	15	−1.3 ± 4.8	0.43 (0.29 to 0.63)	<.001	56	−1.5 ± 3.0	54	0.5 ± 2.2	0.63 (0.34 to 1.17)	.142
Mental fatigue	14	−2.4 ± 4.0	15	−0.9 ± 2.4	0.54 (0.38 to 0.77)	<.001	56	−0.6 ± 1.3	54	0 ± 0.9	0.97 (0.57 to 1.65)	.924
Other late effects												
Sexual functioning	26	7.1 ± 16.4	14	7.1 ± 14.2	0.97^b^ (0.49 to 1.93)	.941	42	−2.4 ± 19.0	55	−0.9 ± 12.6	0.62 (0.41 to 0.94)	.024
Body image	28	18.0 ± 16.4	22	9.6 ± 20.5	1.46 (0.97 to 2.21)	.070	40	0.6 ± 10.4	46	−3.4 ± 12.6	1.31 (0.79 to 2.16)	.291
Breast symptoms	9	−13.6 ± 12.2	8	−4.2 ± 17.9	0.64 (0.35 to 1.18)	.154	61	0.6 ± 0.1	60	0.9 ± 8.7	0.80 (0.56 to 1.15)	.230
Arm symptoms	18	−16.0 ± 20.2	10	3.3 ± 16.6	0.30 (0.13; 0.69)	.005	52	3.8 ± 15.7	59	2.1 ± 10.0	0.86 (0.59 to 1.27)	.454
HRQoL												
Physical functioning	13	7.8 ± 13.4	18	−0.7 ± 10.1	2.28 (1.34 to 3.87)	<.001	57	−0.2 ± 4.3	51	−2.3 ± 6.7	1.39 (0.98 to 1.98)	.067
Emotional functioning	6	18.1 ± 17.8	10	0.3 ± 27.3	2.57 (0.71 to 9.33)	.151	64	0.2 ± 11.1	59	0.2 ± 6.3	1.02 (0.71 to 1.47)	.928
Role functioning	7	31.0 ± 22.4	10	1.7 ± 20.0	2.67 (0.82 to 8.73)	.103	63	3.2 ± 14.9	58	−4.6 ± 16.7	1.36 (0.93 to 1.99)	.107
Social functioning	9	44.4 ± 25.0	9	11.1 ± 16.7	6.95 (2.61 to 18.54)	<.001	61	−1.4 ± 18.3	60	−0.6 ± 15.0	0.98 (0.66 to 1.45)	.909
Cognitive functioning	23	18.8 ± 18.3	24	6.9 ± 13.8	2.49 (1.39 to 4.45)	.002	46	−2.2 ± 14.3	44	1.5 ± 7.9	1.15 (0.75 to 1.78)	.516
Fatigue	18	−17.9 ± 21.9	15	−4.8 ± 18.9	0.35 (0.17 to 0.74)	.006	51	1.1 ± 12.4	54	1.5 ± 14.7	0.86 (0.59 to 1.26)	.449
Nausea and vomiting	8	−12.5 ± 11.8	6	−2.8 ± 12.5	0.31 (0.10 to 1.00)	.049	62	0.8 ± 3.6	63	0.5 ± 2.9	1.02^b^ (0.72 to 1.44)	.898
Pain	23	−15.9 ± 16.3	27	−7.4 ± 17.5	0.59 (0.31 to 1.10)	.098	47	5.7 ± 14.4	42	7.5 ± 15.7	0.96 (0.62 to 1.49)	.859
Dyspnea	19	−21.1 ± 16.5	12	−8.2 ± 15.1	0.44 (0.20 to 0.96)	.040	51	4.7 ± 11.7	56	5.9 ± 12.9	0.90^b^ (0.61 to 1.33)	.606
Insomnia	17	−5.9 ± 21.2	17	7.8 ± 14.6	0.42 (0.17 to 1.03)	.059	53	−2.5 ± 14.4	51	2.0 ± 14.0	0.68 (0.44 to 1.06)	.092
Appetite loss	3	−44.4 ± 19.2	1	−33.3 ± 0	0.26 (0.02 to 3.08)^b^	.283	66	−0.5 ± 9.2	68	0 ± 5.8	0.93 (0.66 to 1.31)	.672
Constipation	4	−41.7 ± 16.7	5	6.7 ± 27.9	0.07 (0.01 to 0.37)	.002	66	−0.5 ± 16.0	64	0 ± 15.7	0.96 (0.67 to 1.38)	.814
Diarrhea	16	−14.6 ± 27.1	21	−6.3 ± 22.7	0.90 (0.42 to 1.90)	.774	54	4.9 ± 13.6	46	1.5 ± 6.9	1.12^b^ (0.75 to 1.67)	.590
Financial difficulties	13	−20.5 ± 16.9	9	−11.1 ± 16.7	0.81 (0.31 to 2.12)	.671	57	3.5 ± 12.1	60	1.1 ± 6.0	1.07^b^ (0.74 to 1.55)	.701

Data are expressed as mean ± SD. *P*-value is derived from a beta regression.

Abbreviations: BCSs = breast cancer survivors; HRQoL = health-related quality of life; QoL = quality of life; T0 = baseline; T1 = post-intervention; T2 = 1-year follow-up.

a Analyses were adjusted for baseline values of the respective outcome variable.

b Not controlled for baseline because all in this subgroup had the same baseline value.

## Discussion

To the best of our knowledge, this is the first RCT to evaluate the effect of aerobic exercise on late effects and HRQoL in long-term BCSs. Our findings demonstrate that such exercise therapy significantly improves fatigue, body image, physical-, role-, (ie, the ability to perform usual work or daily activities) and cognitive function, insomnia, and global health/QoL in BCSs even a decade after treatment cessation. Given that a >10% improvement in the respective outcomes is considered clinically relevant,^34^ aerobic exercise yielded meaningful improvements in total fatigue, physical fatigue, and insomnia. Importantly, exercise therapy was particularly beneficial in BCSs experiencing late effects at baseline. At 1-year follow-up, persistent improvements were only observed for mental fatigue, insomnia, and global health/QoL in the exercise group.

Our findings align with existing evidence documenting that exercise therapy is an effective intervention to reduce fatigue in BCSs.^15^ However, most prior studies have been conducted early in the cancer survivorship continuum. Our findings are also in support of the updated ASCO guidelines for fatigue management,^10^ which strongly recommends exercise therapy to alleviate fatigue severity post-cancer treatment. Notably, the ASCO recommendations are based on moderate-level evidence drawn from merely 9 RCTs. By including long-term BCSs more than a decade after treatment completion, our study expands the evidence base for these recommendations. Long-term cancer survivors represent a critical population to study, as it is a growing population where nearly 30% report chronic fatigue, which likely has a negative impact on their daily functioning and QoL.^6^ Although participants with self-evaluated severe fatigue were excluded from participating in the present trial, 21% of the included BCSs reported chronic fatigue at baseline. Importantly, this subgroup demonstrated substantial improvements following aerobic exercise, with clinically relevant reductions across all fatigue domains.^34^ Hence, future research should focus on the effects of exercise therapy in long-term BCSs with chronic fatigue.

Evidence-based exercise guidelines consistently recommend integrating exercise therapy both during^11^ and after^9^ curative cancer therapies to effectively mitigate treatment-related side effects, such as fatigue and impaired physical function, and to increase HRQoL. However, limited research has hindered the development of exercise therapy guidelines for other late effects, including cognitive function, pain, insomnia, and neuropathy.^9^^,^^35^ A recent review by Sturgeon et al.^35^ aimed to update the exercise guidelines for the 10 understudied outcomes (eg, cognitive function, pain, insomnia, and sexual function) identified by the American College of Sports Medicine in 2019.^9^ Despite the authors’ efforts, insufficient data prevented guideline updates for these outcomes. Thus, our trial contributes to the literature by addressing some of these gaps by demonstrating that aerobic exercise significantly improves cognitive function and insomnia in a large cohort of long-term BCSs. At baseline, 24% of participants reported insomnia, whereas aerobic exercise reduced the odds of insomnia by 39% at post-intervention and by 55% at 1-year follow-up compared to usual care. However, our trial did not observe significant improvements in other late effects, such as breast and arm symptoms or neuropathy, and the small improvement in sexual function observed may not be clinically meaningful. Since our intervention was designed to improve cardiorespiratory fitness through treadmill-based aerobic intervention, it may insufficiently address upper extremity function. Thus, diverse exercise modalities may improve a broader spectrum of late effects more effectively.

Our findings indicate that aerobic exercise among long-term BCSs improves global health/QoL both immediately post-intervention and at 1-year follow-up. However, a slight decline in global health/QoL was observed at the latter time point in the exercise group, indicating that maintaining an active lifestyle over time is likely critical to sustaining high HRQoL, as supported by previous studies.^36^ In our cohort, baseline HRQoL exceeded published population norms, with global health/QoL and functioning scales >90% of age- and sex-specific predictions.^37^ Post-intervention, the exercise group surpassed these levels. The mean scores on the functional EORTC QLQ-C30 scales did, however, range from 32% to 77% among BCSs with low levels of functioning at baseline. In this subgroup, aerobic exercise resulted in clinically relevant improvements across several functional and symptom scores. However, other exercise regimens, such as group-based or concurrent aerobic- and resistance training, may be more effective at enhancing HRQoL than the treadmill-based intervention utilized in the current trial.

### Clinical implications

With the growing number of cancer survivors, the need for effective interventions to support long-term health throughout survivorship is becoming increasingly critical. Our findings in BCSs engaging in exercise intervention an average of >10 years after diagnosis suggest that exercise therapy should be a core component of managing late effects and enhancing HRQoL in BCSs. Notably, the benefits of exercise therapy appeared to be more pronounced among survivors with a higher level of late effects. Hence, personalized exercise interventions that specifically address the unique challenges faced by cancer survivors should be integrated into standard cancer care to optimize survivorship outcomes.

### Limitations

The results presented are secondary analyses from the CAUSE trial,^38^ which was primarily designed to improve cardiorespiratory fitness in long-term BCSs. Consequently, several participants with self-evaluated severe fatigue refrained from participation, likely resulting in the recruitment of BCSs with higher functioning levels and lower symptom burden than the real-world population of BCSs. This may have limited the external validity of our results. Second, the loss to follow-up at T2 may have affected the statistical power of these analyses. Third, results from questionnaires may be subject to reporting bias, which could influence the reliability.

## Conclusion

Aerobic exercise significantly improves fatigue, body image, physical-, role-, and cognitive functioning, insomnia, and HRQoL in long-term BCSs more than a decade after therapy. Importantly, the exercise benefits were more pronounced in BCSs experiencing late effects at baseline than BCSs not experiencing late effects. However, at the 1-year follow-up, most improvements regressed towards baseline values.

## Supplementary Material

pkaf102_Supplementary_Data

## Data Availability

The data underlying this article will be shared on reasonable request to the corresponding author.
