# Supplementary material for: Effects of aerobic exercise on late effects and quality of life in long-term breast cancer survivors: a randomized controlled trial
Source: JNCI Cancer Spectr. 2025 Oct 21;9(6):pkaf102. doi: 10.1093/jncics/pkaf102 (PMC12613246; doi:10.1093/jncics/pkaf102)
Supplement: pkaf102_Supplementary_Data [file pkaf102_supplementary_data.zip › Johansen_et_al.docx._SUPPLEMENT]

**Effects of Aerobic Exercise on Late Effects and Quality of Life in Long-Term Breast Cancer Survivors: A Randomized Controlled Trial**

**Supplemental material**

**S1.** CONSORT Checklist

**S2.** Attendance and adherence to the exercise intervention

**S3.** Baseline characteristics of participants who responded vs. did not respond to the post-intervention (T2) questionnaire

**S4.** Pre and post values for the sub-group analysis

**S5.** CERT Checklist

**S6.** References

**S1. CONSORT Checklist**^1^

| **Section/Topic Item** | **Checklist item no.** | **CONSORT item** | **Extension for NPT trials** | **Page** |
| --- | --- | --- | --- | --- |
| **Title and abstract** |  |  |  |  |
|  | 1a | Identification as a randomized trial in the title |  | Title page |
|  | 1b | Structured summary of trial design, methods, results, and conclusions (for specific guidance see CONSORT for abstracts) | Refer to CONSORT extension for abstracts for NPT trials | NR |
| **Introduction** |  |  |  |  |
| Background and objectives | 2a | Scientific background and explanation of rationale |  | 4 |
|  | 2b | Specific objectives or hypotheses |  | 4 |
| **Methods** |  |  |  |  |
| Trial design | 3a | Description of trial design (such as parallel, factorial) including allocation ratio | When applicable, how care providers were allocated to each trial group | 5 |
|  | 3b | Important changes to methods after trial commencement (such as eligibility criteria), with reasons |  | NA |
| Participants | 4a | Eligibility criteria for participants | When applicable, eligibility criteria for centers and for care providers | 5 |
|  | 4b | Settings and locations where the data were collected |  | 5 |
| Interventions† | 5 | The interventions for each group with sufficient details to allow replication, including how and when they were actually administered | Precise details of both the experimental treatment and comparator | 6 |
|  | 5a |  | Description of the different components of the interventions and, when applicable, description of the procedure for tailoring the interventions to individual participants. | 5-6 |
|  | 5b |  | Details of whether and how the interventions were standardized. | 6 |
|  | 5c. |  | Details of whether and how adherence of care providers to the protocol was assessed or enhanced | NA |
|  | 5d |  | Details of whether and how adherence of participants to interventions was assessed or enhanced | Supplement |
| Outcomes | 6a | Completely defined pre-specified primary and secondary outcome measures, including how and when they were assessed |  | 6-8 |
|  | 6b | Any changes to trial outcomes after the trial commenced, with reasons |  | NA |
| Sample size | 7a | How sample size was determined | When applicable, details of whether and how the clustering by care providers or centers was addressed | 9 |
|  | 7b | When applicable, explanation of any interim analyses and stopping guidelines |  | NA |
| **Randomization** |  |  |  |  |
| Sequence generation | 8a | Method used to generate the random allocation sequence |  | 5 |
|  | 8b | Type of randomization; details of any restriction (such as blocking and block size) |  | 5 |
| Allocation concealment mechanism | 9 | Mechanism used to implement the random allocation sequence (such as sequentially numbered containers), describing any steps taken to conceal the sequence until interventions were assigned |  | 5 |
| Implementation | 10 | Who generated the random allocation sequence, who enrolled participants, and who assigned participants to interventions |  | NR |
| Blinding | 11a | If done, who was blinded after assignment to interventions (for example, participants, care providers, those assessing outcomes) and how | ~~Whether or not those administering co-interventions were blinded to group assignment~~  If done, who was blinded after assignment to interventions (e.g., participants, care providers, those administering co-interventions, those assessing outcomes) and how | 5 |
|  | 11b | If relevant, description of the similarity of interventions | ~~If blinded, method of blinding and description of the similarity of interventions~~ | NA |
|  | 11c |  | If blinding was not possible, description of any attempts to limit bias | 8 |
| Statistical methods | 12a | Statistical methods used to compare groups for primary and secondary outcomes | When applicable, details of whether and how the clustering by care providers or centers was addressed | 9 |
|  | 12b | Methods for additional analyses, such as subgroup analyses and adjusted analyses |  | 9 |
| **Results** |  |  |  |  |
| Participant flow (a diagram is strongly recommended) | 13a | For each group, the numbers of participants who were randomly assigned, received intended treatment, and were analyzed for the primary outcome | The number of care providers or centers performing the intervention in each group and the number of patients treated by each care provider or in each center | Figure 1 |
|  | 13b | For each group, losses and exclusions after randomization, together with reasons |  | Figure 1 |
|  | 13c |  | For each group, the delay between randomization and the initiation of the intervention | NA |
|  | new |  | Details of the experimental treatment and comparator as they were implemented | NA |
| Recruitment | 14a | Dates defining the periods of recruitment and follow-up |  | 10 |
|  | 14b | Why the trial ended or was stopped |  | NA |
| Baseline data | 15 | A table showing baseline demographic and clinical characteristics for each group | When applicable, a description of care providers (case volume, qualification, expertise, etc.) and centers (volume) in each group. | Table 1 |
| Numbers analyzed | 16 | For each group, number of participants (denominator) included in each analysis and whether the analysis was by original assigned groups |  | Figure 1 |
| Outcomes and estimation | 17a | For each primary and secondary outcome, results for each group, and the estimated effect size and its precision (such as 95% confidence interval) |  | Table 2-3 |
|  | 17b | For binary outcomes, presentation of both absolute and relative effect sizes is recommended |  | NA |
| Ancillary analyses | 18 | Results of any other analyses performed, including subgroup analyses and adjusted analyses, distinguishing pre-specified from exploratory |  | Table 3 |
| Harms | 19 | All important harms or unintended effects in each group (for specific guidance see CONSORT for harms) |  | NA |
| Discussion |  |  |  |  |
| Limitations | 20 | Trial limitations, addressing sources of potential bias, imprecision, and, if relevant, multiplicity of analyses | In addition, take into account the choice of the comparator, lack of or partial blinding, and unequal expertise of care providers or centers in each group | 14 |
| Generalizability | 21 | Generalizability (external validity, applicability) of the trial findings | Generalizability (external validity) of the trial findings according to the intervention, comparators, patients, and care providers and centers involved in the trial | 14 |
| Interpretation | 22 | Interpretation consistent with results, balancing benefits and harms, and considering other relevant evidence |  | 14 |
| **Other information** |  |  |  |  |
| Registration | 23 | Registration number and name of trial registry |  | Supplement |
| Protocol | 24 | Where the full trial protocol can be accessed, if available |  | 5 |
| Funding | 25 | Sources of funding and other support (such as supply of drugs), role of funders |  | Title page |

**S2. Attendance and adherence to the exercise intervention**

Tolerability of the intervention was assessed as attendance, exercise dose modifications and loss to follow-up.^2^ Adherence to the exercise prescription was quantified using the relative (exercise) dose intensity^2^. Planned and completed exercise dosages for all exercise sessions were quantified by a modified version of Training Impulse (TRIMP), using planned and completed HR multiplied by the duration of the prescribed training zone (in minutes), and multiplied with a weighting factor to account for the different session types (higher intensity sessions having shorter durations).^3^

|  | **BCSs Exercise Responded at T2 (n=45)** | **BCSs Exercise**  **Did not respond at T2 (n=25)** | **BCSs Usual Care**  **Responded at T2 (n=37)** | **BCSs Usual Care Did not respond at T2 (n=33)** |
| --- | --- | --- | --- | --- |
| Age at survey (years) | 60.5 [55.9-64.2] | 57.7 [53.1-59.5] | 60.6 [54.7-64.8] | 57.6 [54.1-63.2] |
| Living with partner, n (%) |  |  |  |  |
| Yes | 35 (78) | 16 (64) | 21 (64) | 27 (73) |
| No | 10 (22) | 9 (36) | 12 (36) | 10 (27) |
| Education level >13 years, n (%) |  |  |  |  |
| Yes | 28 (62) | 16 (64) | 22 (67) | 27 (73) |
| No | 17 (38) | 9 (36) | 11 (33) | 9 (24) |
| Meet the WHO recommendation for physical activity at T0^a^ |  |  |  |  |
| Yes | 10 (22) | 4 (16) | 5 (15) | 11 (30) |
| No | 27 (60) | 15 (60) | 17 (51) | 23 (62) |
| BMI | 27.1 [24.1-30.3] | 25.4 [23.1-28.4] | 26.2 [24.0-29.1] | 26.1 [23.5-30.2] |

**S3.** Baseline characteristics of participants who responded vs. did not respond to the post-intervention (T2) questionnaire.

Data expressed as n (%) or median [Q1-Q3]. T0 indicates baseline. T2; one-year follow-up.

^a^>150 minutes of moderate physical activity or >75 minutes of vigorous physical activity per week, or an equivalent combination

**Table S4.** Mean values of the outcome variables at T0 and T1 for subgroups experiencing vs. not experiencing late effects.

|  | **BCSs experiencing late effects at T0** | | | | | |  |  | **BCSs not experiencing late effects at T0** | | | | | |
| --- | --- | --- | --- | --- | --- | --- | --- | --- | --- | --- | --- | --- | --- | --- |
|  | n | Exercise group T0 | Exercise group T1 | n | Usual care T0 | Usual care T1 |  |  | n | Exercise group T0 | Exercise group T1 | n | Usual care T0 | Usual care  T1 |
| **Fatigue** |  |  |  |  |  |  |  |  |  |  |  |  |  |  |
| Total fatigue | 14 | 21.4±6.3 | 14.2±7.4 | 15 | 19.2±4.3 | 17.0±5.7 |  |  | 55 | 11.4±2.4 | 9.3±3.4 | 54 | 11.5±2.1 | 12.4±3.3 |
| Mental fatigue | 14 | 7.9±2.4 | 5.3±2.6 | 15 | 6.7±1.7 | 5.8±1.9 |  |  | 55 | 4.4±0.9 | 3.8±1.1 | 54 | 4.4±1.1 | 3.9±1.3 |
| Physical fatigue | 14 | 13.6±4.3 | 8.8±5.2 | 15 | 12.5±3.1 | 11.2±4.2 |  |  | 55 | 7.1±1.8 | 5.4±2.7 | 54 | 7.1±1.3 | 7.8±2.3 |
| **Other late effects** |  |  |  |  |  |  |  |  |  |  |  |  |  |  |
| Sexual functioning | 26 | 0±0 | 7.1±16.4 | 14 | 0±0 | 7.1±14.2 |  |  | 42 | 37.7±20.2 | 35.3±24.5 | 55 | 32.4±15.2 | 32.1±18.2 |
| Body image | 28 | 51.2±23.4 | 69.1±22.5 | 22 | 57.8±19.3 | 67.4±21.3 |  |  | 40 | 86.9±11.0 | 87.5±12.8 | 46 | 88.2±11.2 | 84.8±15.9 |
| Breast symptoms | 9 | 39.5±15.8 | 25.9±17.9 | 8 | 45.1±8.8 | 41.0±18.4 |  |  | 61 | 6.1±9.0 | 6.7±10.6 | 60 | 7.5±9.7 | 8.4±9.8 |
| Arm symptoms | 18 | 48.1±19.4 | 32.1±27.2 | 10 | 45.6±13.3 | 49.8±17.5 |  |  | 52 | 5.8±9.2 | 9.6±14.9 | 59 | 6.4±9.9 | 8.5±12.9 |
| **HRQoL** |  |  |  |  |  |  |  |  |  |  |  |  |  |  |
| Physical functioning | 13 | 70.6±13.3 | 78.5±16.8 | 18 | 69.7±12.6 | 69.0±14.2 |  |  | 57 | 95.6±6.9 | 95.4±6.8 | 51 | 94.7±5.6 | 92.3±7.3 |
| Emotional functioning | 6 | 62.5±4.6 | 80.6±17.2 | 10 | 65.8±2.6 | 66.1±27.5 |  |  | 64 | 91.9±9.0 | 92.1±11.2 | 59 | 94.5±7.7 | 94.8±8,7 |
| Role functioning | 7 | 31.0±17.8 | 61.9±23.0 | 10 | 41.7±14.2 | 43.3±25.1 |  |  | 63 | 91.9±14.0 | 94.2±12.0 | 58 | 94.0±11.6 | 89.4±17.9 |
| Social functioning | 9 | 27.8±20.4 | 72.2±26.4 | 9 | 27.8±16.7 | 38.9±25.0 |  |  | 61 | 88.8±14.8 | 87.4±21.0 | 60 | 90.0±13.8 | 89.4±15.9 |
| Cognitive functioning | 23 | 48.6±20.7 | 67.4±22.7 | 24 | 56.3±14.6 | 63.2±18.4 |  |  | 46 | 92.0±8.4 | 89.9±13.4 | 44 | 94.3±8.0 | 92.8±10.4 |
| Fatigue | 18 | 62.3±19.5 | 44.4±25.6 | 15 | 63.7±13.6 | 58.9±18.8 |  |  | 51 | 14.8±12.5 | 15.9±17.0 | 54 | 17.6±13.0 | 19.1±15.3 |
| Nausea & vomiting | 8 | 18.8±5.8 | 6.3±8.6 | 6 | 25.0±13.9 | 22.2±13.6 |  |  | 62 | 0±0 | 0.8±3.6 | 63 | 0±0 | 0.5±2.9 |
| Pain | 23 | 42.8±13.1 | 26.8±20.6 | 27 | 50.6±24.2 | 43.2±28.2 |  |  | 47 | 3.2±6.6 | 8.9±15.9 | 42 | 4.4±7.4 | 11.9±16.2 |
| Dyspnea | 19 | 40.4±14.0 | 19.3±20.2 | 12 | 41.7±15.1 | 33.3±20.1 |  |  | 51 | 0±0 | 3.3±10.0 | 56 | 0±0 | 5.9±12.9 |
| Insomnia | 17 | 76.5±15.7 | 45.1±31.0 | 17 | 80.4±16.9 | 64.7±34.3 |  |  | 53 | 18.2±16.8 | 14.5±17.9 | 52 | 19.9±16.5 | 23.1±26.6 |
| Appetite Loss | 3 | 66.7±0.0 | 22.2±19.2 | 1 | 66.7±0 | 33.3±0 |  |  | 66 | 3.0±9.7 | 2.5±8.9 | 68 | 2.5±8.8 | 2.5±8.8 |
| Constipation | 4 | 83.3±19.2 | 41.7±16.7 | 5 | 73.3±14.9 | 7.6±15.2 |  |  | 66 | 8.1±14.4 | 7.6±15.2 | 64 | 8.9±14.8 | 8.9±17.1 |
| Diarrhea | 16 | 39.6±18.1 | 25.0±19.2 | 21 | 36.5±10.0 | 30.2±23.3 |  |  | 54 | 0±0 | 4.9±13.5 | 46 | 0±0 | 1.5±6.9 |
| Financial difficulties | 13 | 41.0±20.0 | 20.5±29.0 | 9 | 59.3±27.8 | 48.1±37.7 |  |  | 57 | 0±0 | 3.5±12.1 | 60 | 0±0 | 1.1±6.0 |

Data expressed as mean±SD. T0 indicates baseline; T1, post-intervention. HRQoL, health-related quality of life.

**S5. CONSORT Checklist**^1^

| **Section/Topic Item** | **Checklist item no.** | **CONSORT item** | **Extension for NPT trials** | **Page** |
| --- | --- | --- | --- | --- |
| **Title and abstract** |  |  |  |  |
|  | 1a | Identification as a randomized trial in the title |  | Title page |
|  | 1b | Structured summary of trial design, methods, results, and conclusions (for specific guidance see CONSORT for abstracts) | Refer to CONSORT extension for abstracts for NPT trials | NR |
| **Introduction** |  |  |  |  |
| Background and objectives | 2a | Scientific background and explanation of rationale |  | 4 |
|  | 2b | Specific objectives or hypotheses |  | 4 |
| **Methods** |  |  |  |  |
| Trial design | 3a | Description of trial design (such as parallel, factorial) including allocation ratio | When applicable, how care providers were allocated to each trial group | 5 |
|  | 3b | Important changes to methods after trial commencement (such as eligibility criteria), with reasons |  | NA |
| Participants | 4a | Eligibility criteria for participants | When applicable, eligibility criteria for centers and for care providers | 5 |
|  | 4b | Settings and locations where the data were collected |  | 5 |
| Interventions† | 5 | The interventions for each group with sufficient details to allow replication, including how and when they were actually administered | Precise details of both the experimental treatment and comparator | 6 |
|  | 5a |  | Description of the different components of the interventions and, when applicable, description of the procedure for tailoring the interventions to individual participants. | 5-6 |
|  | 5b |  | Details of whether and how the interventions were standardized. | 6 |
|  | 5c. |  | Details of whether and how adherence of care providers to the protocol was assessed or enhanced | NA |
|  | 5d |  | Details of whether and how adherence of participants to interventions was assessed or enhanced | Supplement |
| Outcomes | 6a | Completely defined pre-specified primary and secondary outcome measures, including how and when they were assessed |  | 6-8 |
|  | 6b | Any changes to trial outcomes after the trial commenced, with reasons |  | NA |
| Sample size | 7a | How sample size was determined | When applicable, details of whether and how the clustering by care providers or centers was addressed | 9 |
|  | 7b | When applicable, explanation of any interim analyses and stopping guidelines |  | NA |
| **Randomization** |  |  |  |  |
| Sequence generation | 8a | Method used to generate the random allocation sequence |  | 5 |
|  | 8b | Type of randomization; details of any restriction (such as blocking and block size) |  | 5 |
| Allocation concealment mechanism | 9 | Mechanism used to implement the random allocation sequence (such as sequentially numbered containers), describing any steps taken to conceal the sequence until interventions were assigned |  | 5 |
| Implementation | 10 | Who generated the random allocation sequence, who enrolled participants, and who assigned participants to interventions |  | NR |
| Blinding | 11a | If done, who was blinded after assignment to interventions (for example, participants, care providers, those assessing outcomes) and how | ~~Whether or not those administering co-interventions were blinded to group assignment~~  If done, who was blinded after assignment to interventions (e.g., participants, care providers, those administering co-interventions, those assessing outcomes) and how | 5 |
|  | 11b | If relevant, description of the similarity of interventions | ~~If blinded, method of blinding and description of the similarity of interventions~~ | NA |
|  | 11c |  | If blinding was not possible, description of any attempts to limit bias | 8 |
| Statistical methods | 12a | Statistical methods used to compare groups for primary and secondary outcomes | When applicable, details of whether and how the clustering by care providers or centers was addressed | 9 |
|  | 12b | Methods for additional analyses, such as subgroup analyses and adjusted analyses |  | 9 |
| **Results** |  |  |  |  |
| Participant flow (a diagram is strongly recommended) | 13a | For each group, the numbers of participants who were randomly assigned, received intended treatment, and were analyzed for the primary outcome | The number of care providers or centers performing the intervention in each group and the number of patients treated by each care provider or in each center | Figure 1 |
|  | 13b | For each group, losses and exclusions after randomization, together with reasons |  | Figure 1 |
|  | 13c |  | For each group, the delay between randomization and the initiation of the intervention | NA |
|  | new |  | Details of the experimental treatment and comparator as they were implemented | NA |
| Recruitment | 14a | Dates defining the periods of recruitment and follow-up |  | 10 |
|  | 14b | Why the trial ended or was stopped |  | NA |
| Baseline data | 15 | A table showing baseline demographic and clinical characteristics for each group | When applicable, a description of care providers (case volume, qualification, expertise, etc.) and centers (volume) in each group. | Table 1 |
| Numbers analyzed | 16 | For each group, number of participants (denominator) included in each analysis and whether the analysis was by original assigned groups |  | Figure 1 |
| Outcomes and estimation | 17a | For each primary and secondary outcome, results for each group, and the estimated effect size and its precision (such as 95% confidence interval) |  | Table 2-3 |
|  | 17b | For binary outcomes, presentation of both absolute and relative effect sizes is recommended |  | NA |
| Ancillary analyses | 18 | Results of any other analyses performed, including subgroup analyses and adjusted analyses, distinguishing pre-specified from exploratory |  | Table 3 |
| Harms | 19 | All important harms or unintended effects in each group (for specific guidance see CONSORT for harms) |  | NA |
| Discussion |  |  |  |  |
| Limitations | 20 | Trial limitations, addressing sources of potential bias, imprecision, and, if relevant, multiplicity of analyses | In addition, take into account the choice of the comparator, lack of or partial blinding, and unequal expertise of care providers or centers in each group | 14 |
| Generalizability | 21 | Generalizability (external validity, applicability) of the trial findings | Generalizability (external validity) of the trial findings according to the intervention, comparators, patients, and care providers and centers involved in the trial | 14 |
| Interpretation | 22 | Interpretation consistent with results, balancing benefits and harms, and considering other relevant evidence |  | 14 |
| **Other information** |  |  |  |  |
| Registration | 23 | Registration number and name of trial registry |  | Supplement |
| Protocol | 24 | Where the full trial protocol can be accessed, if available |  | 5 |
| Funding | 25 | Sources of funding and other support (such as supply of drugs), role of funders |  | Title page |

**S5. CERT Checklist**^4^

| Section/Topic | Item # | Checklist item | Location** | |
| --- | --- | --- | --- | --- |
|  |  |  | Primary paper (page, table, appendix) | † Other (paper or protocol, website  (URL) |
| WHAT: materials | 1 | Detailed description of the type of exercise equipment (e.g. weights, exercise equipment  such as machines, treadmill, bicycle ergometer etc) | Pg. 6 |  |
| WHO: provider | 2 | Detailed description of the qualifications, teaching/supervising expertise, and/or training  undertaken by the exercise instructor |  | https://pubmed.ncbi.nlm.nih.gov/36920460/ |
| HOW: delivery | 3 | Describe whether exercises are performed individually or in a group |  | https://pubmed.ncbi.nlm.nih.gov/36920460/ |
|  | 4 | Describe whether exercises are supervised or unsupervised and how they are delivered | Pg. 6 |  |
|  | 5 | Detailed description of how adherence to exercise is measured and reported | Supplement |  |
|  | 6 | Detailed description of motivation strategies | NR |  |
|  | 7 a | Detailed description of the decision rule(s) for determining exercise progression |  | https://pubmed.ncbi.nlm.nih.gov/36920460/ |
|  | 7 b | Detailed description of how the exercise program was progressed |  | https://pubmed.ncbi.nlm.nih.gov/36920460/ |
|  | 8 | Detailed description of each exercise to enable replication (e.g. photographs, illustrations ,  video etc | NA |  |
|  | 9 | Detailed description of any home program component (e.g. other exercises, stretching etc) | NA |  |
|  | 10 | Describe whether there are any non-exercise components (e.g. education, cognitive  behavioural therapy, massage etc) | NA |  |
|  | 11 | Describe the type and number of adverse events that occurred during exercise | NA |  |
|  | 12 | Describe the setting in which the exercises are performed |  | https://pubmed.ncbi.nlm.nih.gov/36920460/ |
|  | 13 | Detailed description of the exercise intervention including, but not limited to, number of  exercise repetitions/sets/sessions, session duration, intervention/program duration etc |  | https://pubmed.ncbi.nlm.nih.gov/36920460/ |
|  | 14 a | Describe whether the exercises are generic (one size fits all) or tailored whether tailored to the individual | Pg. 6 |  |
|  | 14 b | Detailed description of how exercises are tailored to the individual | Pg. 6 | https://pubmed.ncbi.nlm.nih.gov/36920460/ |
|  | 15 | Describe the decision rule for determining the starting level at which people commence an exercise program (such as beginner, intermediate, advanced etc) | NA |  |
|  | 16 a | Describe how adherence or fidelity to the exercise intervention is assessed/measured | Supplement | https://pubmed.ncbi.nlm.nih.gov/36920460/ |
|  | 16 b | Describe the extent to which the intervention was delivered as planned | Pg. 10 |  |

S6. References

1. Boutron I, Altman DG, Moher D, Schulz KF, Ravaud P, CONSORT NPT Group. CONSORT Statement for Randomized Trials of Nonpharmacologic Treatments: A 2017 Update and a CONSORT Extension for Nonpharmacologic Trial Abstracts. Ann Intern Med. 2017;167(1):40–7.

2. Nilsen TS, Scott JM, Michalski M, Capaci C, Thomas S, Herndon JE, et al. Novel Methods for Reporting of Exercise Dose and Adherence: An Exploratory Analysis. Med Sci Sports Exerc. 2018;50(6):1134–41.

3. Hayes PR, Quinn MD. A mathematical model for quantifying training. Eur J Appl Physiol. 2009;106(6):839–47.

4. Slade SC, Dionne CE, Underwood M, Buchbinder R. Consensus on Exercise Reporting Template (CERT): Explanation and Elaboration Statement. Br J Sports Med. 2016;50(23):1428–37.
